# Local genetic ancestry in *CDKN2B-AS1* is associated with primary open-angle glaucoma in an African American cohort extracted from de-identified electronic health records

**DOI:** 10.1186/s12920-018-0392-4

**Published:** 2018-09-14

**Authors:** Nicole A. Restrepo, Sarah M. Laper, Eric Farber-Eger, Dana C. Crawford

**Affiliations:** 1 Department of Population and Quantitative Health Sciences, Institute for Computational Biology, Case Western Reserve University, 2103 Cornell Road, Wolstein Research Building, Suite 2-527, Cleveland, OH 44106 USA; 2 Eastern Virginia Medical School, Norfolk, VA USA; 3 Vanderbilt Institute for Clinical and Translational Research, Vanderbilt University Medical Center, Nashville, TN USA

**Keywords:** African Americans, Primary open-angle glaucoma, Electronic health records

## Abstract

**Background:**

Glaucoma is a leading cause of blindness in developed countries. Primary open-angle glaucoma (POAG), the most prevalent clinical subtype of glaucoma in the United States, affects African Americans at a higher rate compared with European Americans. Risk factors identified for POAG include increased age and family history, which coupled with heritability estimates, suggest this complex condition is associated with genetic and environmental factors. To date, several genome-wide studies have identified loci significantly associated with POAG risk, but most of these studies were performed in populations of European-descent.

**Methods:**

To identify population-specific and trans-population genetic associations for POAG, we genotyped 11,521 African Americans using the Illumina Metabochip as part of the Epidemiologic Architecture for Genes Linked to Environment (EAGLE) study accessing BioVU, the Vanderbilt University Medical Center’s biorepository linked to de-identified electronic health records. Among this study population, we identified 138 cases of POAG and 1376 controls and performed Metabochip-wide tests of association. We also estimated local genetic ancestry at *CDKN2B-AS1*, a POAG-associated locus established in European-descent populations.

**Results:**

Overall, we did not identify significant single SNP-POAG associations after adjusting for multiple testing. We did, however, detect a significant association between POAG risk and local African genetic ancestry at *CDKN2B-AS1*, where on average cases were of 90% African descent compared with controls at 58% (*p* = 2 × 10^− 6^).

**Conclusions:**

These data suggest that *CDKN2B-AS1* is an important locus for POAG risk among African Americans, warranting further investigation to identify the variants underlying this association.

**Electronic supplementary material:**

The online version of this article (10.1186/s12920-018-0392-4) contains supplementary material, which is available to authorized users.

## Background

Glaucoma is the second leading cause of blindness in the United States, and it is the leading cause of blindness and irreversible vision loss in African Americans [1], with a prevalence approximately double that observed in European-descent populations [1–3]. The prevalence of glaucoma is similar for European, Japanese, and Indian populations with rates approaching those observed in African descent populations in the oldest age categories [4]. Although African Americans comprise the group of highest risk of developing glaucoma-related vision problems, many cases remain undiagnosed. Previous studies have suggested that nation-wide implementation of screening middle aged African Americans could decrease the rate of undiagnosed glaucoma from 50 to 27% [5]. Earlier screening and diagnosis enables patients to more effectively leverage current treatment options to reduce the risk of bilateral blindness later in life [5].

In addition to African ancestry and age [6], other known risk factors associated with the development of glaucoma include myopia [7] and high intraocular pressure [6, 8, 9]. Family history has also been associated with glaucoma risk [6, 10, 11], albeit inconsistently most likely due to the heterogeneous nature of the disease. The phenotypic heterogeneity of glaucoma has also impacted other studies attempting to establish and quantify the genetic contribution to risk in developing the disease; consequently, the majority of these studies have been conducted on more easily-measured glaucoma endophenotypes such as central corneal thickness (h^2^ = 0.35–0.72%) [12–14], intraocular pressure (h^2^ = 0.35–0.94%) [12, 14], and cup-to-disc ratio (h^2^ = 0.56–0.66%) [12, 15]. Pulsatility of choroidal blood flow and velocity are additional quantitative traits whose variation from normal parameters has been observed in individuals with glaucoma [16, 17], yet heritability studies have not yet found significant genetic contribution to its variability [12].

The strongest evidence for a genetic contribution related to glaucoma comes from studies of primary open-angle glaucoma (POAG), the most prevalent clinical subtype of glaucoma in the United States. Early linkage and family-based genetic association studies identified the *MYOC* (myocilin)*, OPTN* (optineurin)*,* and *WDR36* (WD repeat domain 36) [18–20] genes as the primary genes for susceptibility to POAG. Mutations in *MYOC* are known to cause hereditary early-onset POAG in multiple populations [18, 21, 22]. More recently, large-scale genome-wide association studies (GWAS) have identified variants in the *CAV1/CAV2*, *CDKN2B-AS1* and *SIX1/SIX6I* genes that influence POAG risk in European-descent and Japanese populations [23–27].

Additional genetic factors that have yet to be discovered are hypothesized to drive POAG risk and to account for the differences in incidence observed across racial/ethnic groups. For example, in a study of African Americans, the frequency of *MYOC* mutations was comparably lower (~ 1.4%) than in other populations (~ 2–4%) [28] suggesting that other genetic loci are driving risk in this group. It is possible that both population-specific and trans-population genetic variants contribute to POAG risk. To identify population-specific and trans-population genetic factors, we conducted a hypothesis-testing and hypothesis-generating genetic association study in African Americans with and without POAG drawn from a clinical cohort with electronic health records.

## Methods

### Study population and genotyping

The study population is a subset of the Epidemiologic Architecture for Genes Linked to Environment (EAGLE) study, a study site of the larger Population Architecture using Genomics and Epidemiology (PAGE) I study [29, 30]. In general, the PAGE study is a consortium of diverse epidemiologic and clinical cohorts with broad research goals that include the generalization of genetic associations to multiple populations [29]. To identify non-European Americans for PAGE I, EAGLE accessed the Vanderbilt University Medical Center (VUMC)‘s biorepository linked to de-identified electronic health records known as BioVU [31].

VUMC’s BioVU followed an opt-out model for DNA sample accrual between 2007 and 2015 [31]. That is, DNA was collected from discarded blood samples remaining after routine clinical testing and was linked to de-identified electronic health records. According to the Vanderbilt Institutional Review Board (IRB) and the Federal Office of Human Research Protections provisions, this VUMC protocol is considered nonhuman subjects research (The Code of Federal Regulations, 45 CFR 46.102 (f)) [31, 32].

As previously described [33], EAGLE selected all non-European Americans from BioVU as of 2011 for genotyping on the Metabochip (EAGLE BioVU). A total of 11,521 African Americans samples in EAGLE BioVU were genotyped [33]. From among these patients, billing and procedural codes along with text searches were used to identify POAG cases (*n* = 138) and controls (*n* = 1376). In short, controls included patients in BioVU over the age of 60 years whose records did not contain an ICD-9-CM code for any form of glaucoma nor any mention of “glaucoma” in a text search of their ‘Problems List.’ Manual review of all cases and a subset of controls was performed for quality assurance as previously described [34].

The Metabochip is an Illumina (San Diego, CA) custom array designed for fine-mapping of metabolic and cardiovascular traits. Fine-mapping regions cover 257 loci chosen from SNPs that reached genome-wide significance from select consortium meta-analyses [35]. The Metabochip was also designed for replication of GWAS-identified index variants for any phenotype from the GWAS Catalog (http://www.ebi.ac.uk/gwas/) as of 2009. A total of 33 GWAS-index variants representing ocular diseases (including age-related macular degeneration, POAG, normal tension glaucoma, and diabetic retinopathy) as well as related traits (myopia, ocular axial length, HbA1c, cup-to-disc ratio, intraocular pressure, and optic disc size) are directly assayed by the Metabochip (Additional file 1: Table S1).

EAGLE BioVU DNA samples were genotyped using the Metabochip following the manufacturer’s protocol (Illumina, Inc.; San Diego, CA.), and 360 HapMap samples, including YRI samples, were genotyped for PAGE-wide cross-study quality control standards [36]. A description of the genotyping protocols and quality control measures has been previously published [30]. In brief, genetic variants were evaluated for deviations from Hardy Weinberg Equilibrium, which may be a result of poor genotyping. Variants with a genotyping call rate < 95% were removed from further analysis. Principal components (PC) were calculated using EIGENSOFT [37, 38]. At the sample level, DNA samples with poor sample call rate (< 95%), sex discordance, or evidence of cryptic relatedness were removed from analyses.

### Statistical methods

Individuals included in this analysis were those identified as POAG cases over the age of 20 years and POAG controls over the age of 60 years. An older age threshold was applied in controls to minimize the probability of including potential future cases. African Americans are at increased risk of glaucoma over the age of 40 years, while other populations have an age-associated risk over 60 years. Age was defined as age at diagnosis in cases and age at last clinical exam in controls. T-tests and chi-square tests, where appropriate, were used to compare demographic clinical characteristics between cases and controls in Stata/SE version 14.2.

All common variants (MAF > 0.05) were tested for an association with POAG using logistic regression separately assuming a log-additive genetic model (Additional file 1: Table S2), a recessive model (Additional file 1: Table S3), and a dominant model (Additional file 1: Table S4) adjusted by age, sex, the first three PCs, and median diastolic blood pressure. Analyses were conducted using PLINKv1.90 [39]. Additionally, we tested for an association between POAG and 258 SNPs that passed quality control in the *CDKN2B-AS1* region of chromosome 14. Pair-wise linkage disequilibrium (r^2^) was calculated in SNAP [40] using YRI 1000 Genomes Project Pilot 1 reference data. Power calculations were performed in Quanto [41] to determine 80% power to detect an association with a case:control ratio of 1:3, assuming a log-additive model and a genome-wide significance threshold (5 × 10^− 8^).

### Local ancestry mapping

Local ancestry for the *CDKN2B-AS1* region, located on chromosome 9, was determined for the POAG cases and controls using Local Ancestry in adMixed Populations (LAMP) [42]. Input parameters included the estimated number of generations since admixture (generations = 10), estimated fraction of admixture from each population (African = 0.8, European = 0.2), and predicted recombination rate (3.4 × 10^− 7^ bases^− 1^). The number of alleles from the ancestral populations at each SNP that was genotyped in this region was estimated, and the overall fraction of alleles from each ancestral population in this region for each patient was determined. Percent African ancestry was then tested for an association with POAG status using logistic regression using R software version 3.1.3 [43].

### Global ancestry mapping

Global ancestry was calculated using fastSTRUCTURE [44] with all of the Metabochip data. Input parameters were set to default, as recommended by the authors, and the analysis was set to determine the proportion of two populations (K = 2). Admixture plots were graphed using the web graphical interface (http://pophelper.com/) of the R module “pophelper” [45].

## Results

### Population characteristics

A total of 138 African American POAG cases and 1376 controls passed quality control in EAGLE BioVU for the present study. We previously described [34] these cases compared with 4813 controls over the age of 40; in the present study, we compare the same cases with subset of the controls over the age of 60 years (Table 1). Here, cases were younger (*p* = 0.01) with higher cholesterol levels (183 mg/dL; *p* = 0.01), more likely to be female with higher average body mass index (30.1 kg/m^2^) in comparison to controls (28.8 kg/m^2^ and 169 mg/dL, respectively). Cases also presented with higher triglyceride levels compared to controls (125 mg/dL versus 97 mg/dL; *p* = 0.0001).

**Table 1:** Study population characteristics of primary open-angle glaucoma cases and controls among African Americans in EAGLE BioVU

Trait	Cases (*n* = 138)	Controls (1376)	*p*-value
Median age at diagnosis or LCV (years)	62.0 (12.0)	67.3 (7.8)	0.01
% female	63.7	56.5	0.10
% hypertensive	55.1	52.5	0.50
Median BMI (kg/m^2^)	30.1 (6.7)	28.8 (7.35)	0.44
Median diastolic blood pressure (mm/Hg)	74.5 (8.1)	76.0 (8.8)	0.97
Median systolic blood pressure (mm/Hg)	134.5 (14.1)	135 (14.6)	0.88
Median cholesterol (mg/dL)	183 (40.6)	169 (46.7)	0.01
Median HDL-C (mg/dL)	52.5 (25.0)	49 (17.8)	0.88
Median LDL-C (mg/dL)	103 (42.9)	93 (37.4)	0.20
Median triglycerides (mg/dL)	125 (76.3)	97 (68.1)	0.0001

Case extraction was described in Restrepo et al. [34]. Control extraction from EAGLE BioVU was also described in Restrepo et al. [34] but restricted to controls > 40 years of age (as opposed to > 60 years of age here). Values were defined or calculated for the following: Age at POAG diagnosis was determined by the date of when the POAG billing code (ICD-9-CM 365.11) was first mentioned in the records. Age at last clinic visit (LCV) was taken as the date of the last current procedure terminology (CPT) code mentioned in the records for controls. An individual was classified as hypertensive if he/she met one of three criteria: systolic blood pressure > 140 mm/Hg, diastolic blood pressure > 90 mm/Hg, or on hypertension medications all within a 2-year window of when he or she was diagnosed with POAG for cases and a 2-year window of his or her LCV date for controls. Median (and standard deviation) blood pressure (systolic and diastolic), lipids (total cholesterol, high-density lipoprotein cholesterol or HDL-C, low-density lipoprotein cholesterol or LDL-C, and triglycerides), and body mass index (height and weight) were calculated from all labs or measurements available within 2 years of POAG diagnosis or LCV. T-tests and chi-square tests, where appropriate, were used to compare demographic clinical characteristics between cases and controls. Abbreviations: standard deviation (SD)

### Local ancestry in the *CDKN2B-AS1* region

We previously reported on preliminary tests of association in the *CDKN2B-AS1* region [34], which was fine-mapped by the Illumina Metabochip. None of the tests of association were significant after correcting for the 258 common variants tested [34]. As we have already noted, there are multiple explanations for the lack of significant results including limited power, variability in linkage disequilibrium patterns across populations, and genetic heterogeneity. Another possible explanation for the observed null results is that the previous analysis did not account for local genetic ancestry. We therefore sought to determine whether the total composition of African and European ancestry at this region could account for POAG risk.

**Fig. 1:**
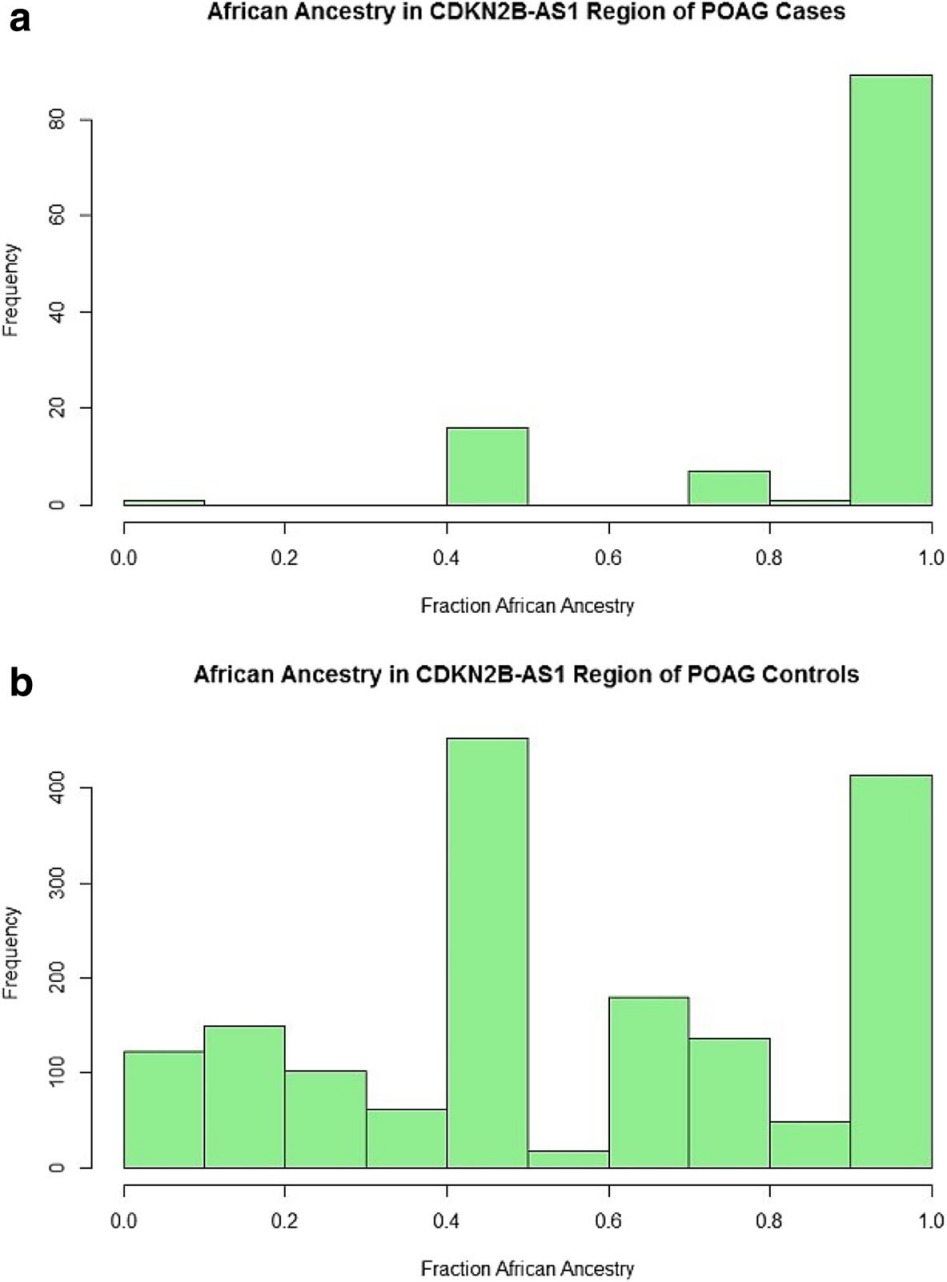
Distribution of African ancestry at *CDKN2B-AS1* in African American primary open-angle glaucoma cases and controls from EAGLE BioVU. Fraction of African ancestry, estimated by LAMP using genotype data available for *CDKN2B-AS1* from the Illumina Metabochip, is plotted on the x-axis with frequency on the y-axis for primary open-angle glaucoma (POAG) **a** cases (*n* = 138) and **b** controls (*n* = 1376). Plots were graphed in R

Local ancestry for the *CDKN2B-AS1* region was determined for POAG cases and controls using LAMP [42]. The number of alleles from the ancestral populations at each SNP was estimated, and the overall fraction of alleles from each ancestral population in this region for each patient was calculated. Logistic regression was then performed between POAG case status and percent African ancestry to assess whether ancestry might alter POAG risk. The mean African ancestry for POAG cases and controls at *CDKN2B-AS1* was 0.90 and 0.58, respectively (Fig. 1), and the percent of African ancestry in the *CDKN2B-AS1* region was significantly associated with POAG at *p* = 2 × 10^− 6^. In contrast, the average Metabo-wide global African ancestry for cases and controls was 81.5 and 79.4%, respectively, in agreement with previous estimates [46, 47].

### Metabochip-wide association of POAG in African Americans

**Fig. 2:**
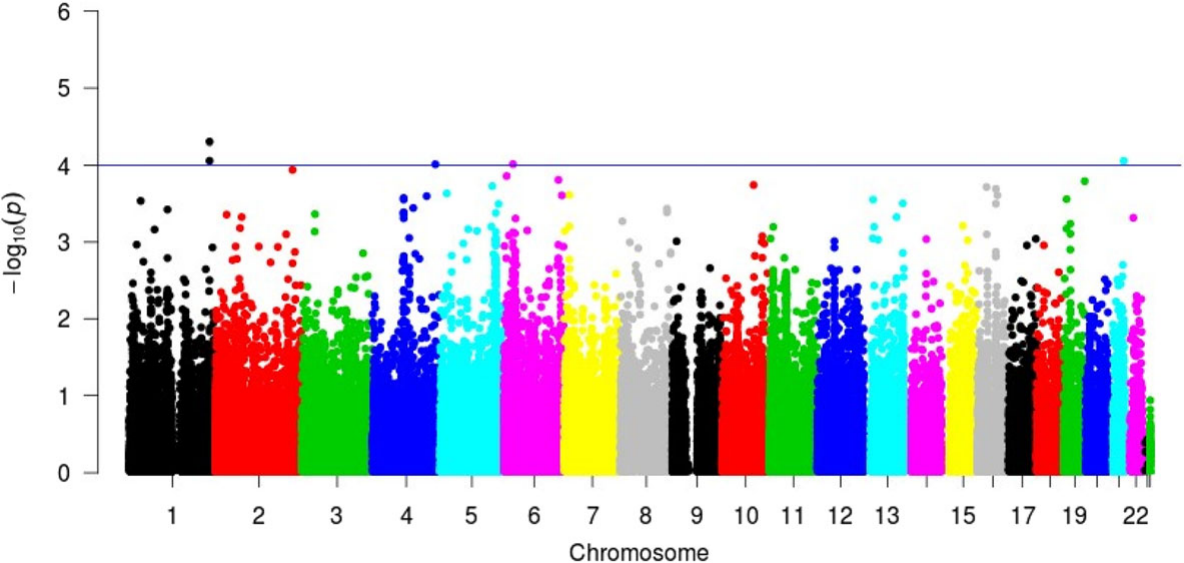
Manhattan plot of EAGLE BioVU primary open-angle glaucoma Metabochip-wide tests of association in African Americans. Logistic regression assuming an additive genetic model was performed for 138 cases and 1376 controls adjusted by age, sex, principal components, and median diastolic blood pressure. *P*-values [(−log_10_) on the y-axis] for each test of association are plotted by chromosome (x-axis). The blue line depicts a suggestive significance threshold of *p* < 5.0 × 10^− 4^

We tested all SNPs genotyped on the Illumina Metabochip for an association with POAG adjusted for age, sex, the first three principal components, and median diastolic blood pressure (Fig. 2; Additional file 1: Tables S2-S4). No SNP was significantly associated with POAG after adjusting for a strict Bonferroni correction (*p* < 4.04 × 10^− 7^). The two most significant associations [chr1:228347779 (rs4846835) and chr1:228354829 (rs34783939)] under the log-additive genetic model are located within the protein coding gene of *GALNT2*, a member of the glycosyltransferase 2 protein family. *GALNT2* was targeted for fine-mapping by the Metabochip based on earlier reported associations with high density lipoprotein cholesterol (HDL-C) and triglyceride levels [48, 49]. It is interesting to note that *GALNT2* rs4846835 was associated with dementia and core Alzheimer’s disease neuropathologic changes, albeit not at the genome-wide level [50]. Both of these variants also appear as marginally significant under a dominant genetic model (Table 3 [OR = 2.43 & 2.24 respectively]. Homozygous carriers for either of the two SNPs are rare in cases and controls at only a frequency of 1 to 2%. Variant rs4846835 heterozygotes account for 9.4% of cases and 17.5% of controls, while rs34783939 heterozygotes make up 12.3% of cases and 23.6% of controls. The variants are not in strong linkage disequilibrium with one another (*r*^2^ = 0.304 in YRI, phase 1 1000 Genomes Project). It is important to note that rs34783939 is most likely multi-allelic based on later versions of the 1000 Genomes Project and other large-scale sequencing efforts.

Additional variants of interest for future studies that were marginally significant in both additive and dominant genetic modes are rs13423742 (OR_additive_ = 3.04; *p* = 1.14 × 10^− 4^), rs9479726 (OR_additive_ = 0.41; *p* = 1.54 × 10^− 4^), and rs1671152 (OR_additive_ = 1.91; *p* = 1.6 × 10^− 4^). Variant rs1671152 is a known missense variant in the glycoprotein VI (*GP6*) gene. *GP6*, a collagen receptor, is involved in platelet aggregation [54]. *GP6* RNAs are expressed in the retina and brain as shown in the FANTOM5 and GTEx datasets [55, 56]. In 1000 Genomes Project phase 3 CEU the rs1671152 (A) allele has a frequency of 0.182 while the YRI population has a frequency of 0.324, consistent with that observed in EAGLE BioVU African Americans (coded allele frequency = 0.32). The frequency of rs1671152 is twice as high in African Americans compared with European Americans suggesting it could potentially be a population-specific factor.

The intergenic SNP rs9479726 was less frequent in cases compared with controls (OR_additive_ = 0.41; 1.54 × 10–4 and OR_dominant_ = 0.42; 2.5 × 10^− 4^). None of the cases were found to be homozygous for the coded allele, while 5.7% of controls were homozygous. The ‘A’ allele had a frequency of 24% in the overall population, with 10.8% of cases and 37.6% of controls being heterozygous carriers.

One SNP (i.e., rs7454156) was consistently associated with POAG at *p* < 5.0 × 10^− 4^ in both the additive (Table 2) and recessive genetic models (Table 3). This intronic variant (G) in the bone morphogenetic protein 6 (*BMP6*) was found to be homozygous in 5% of controls and 2.5% of cases. In a mouse model of hemochromatosis, mutations in a BMP6 co-receptor (i.e., *HJV*) were found to result in an accumulation of iron in mouse retinal tissues and upregulation of *BMP6* along with upregulation of *VEGF* that resulted in subsequent abnormal vascularization of the retina [57].

**Table 2:** Ten most significant results for primary open-angle glaucoma Metabochip-wide genetic associations in African Americans

CHR	SNP	Gene	CA	CAF	OR	95% CI	*p*-value
1	rs4846835	*GALNT2*	A	0.11	2.37	1.56–3.60	5.00 × 10^− 5^
1	rs34783939	*GALNT2*	C	0.15	2.09	1.44–3.02	8.73 × 10^− 5^
21	rs9982695	*C21orf33*	A	0.24	2.09	1.44–3.02	8.74 × 10^− 5^
4	rs3775202	*VEGFC*	G	0.43	1.92	1.38–2.66	9.70 × 10^−5^
2	rs13423742	*FN1*	C	0.06	3.04	1.73–5.36	1.14 × 10^− 4^
6	rs7454156	*BMP6*	G	0.18	2.08	1.42–3.02	1.37 × 10^− 4^
6	rs9479726	*RGS17-OPRM1*	A	0.24	0.41	0.25–0.64	1.54 × 10^−4^
19	rs1671152	*GP6*	A	0.32	1.91	1.36–2.68	1.60 × 10^−4^
10	rs286489	*LOC101929727*	A	0.28	1.90	1.35–2.66	1.80 × 10^−4^
5	rs4336354	*HTR4*	G	0.09	2.51	1.54–4.07	1.86 × 10^−4^

Logistic regression assuming an additive genetic model was performed for 138 cases and 1376 controls adjusted by age, sex, principal components, and median diastolic blood pressure. For the ten most significant associations, chromosome (CHR), SNP ID (rs number), gene, coded allele (CA), coded allele frequency (CAF), odds ratio (OR), 95% confidence interval (CI), and *p*-value are given

**Table 3:** Primary open-angle glaucoma Metabochip-wide genetic associations in African Americans for dominant and recessive models that overlap with the most significant results for the additive genetic model

CHR	SNP	Gene	CA	OR	95% CI	*p*-value	Genetic model
1	rs4846835	*GALNT2*	A	2.43	1.56–3.74	6.59 × 10^−5^	dominant
1	rs34783939	*GALNT2*	C	2.24	1.47–3.39	1.6 × 10^−4^	dominant
2	rs13423742	*FN1*	C	2.82	1.63–4.86	2.02 × 10^−4^	dominant
6	rs9479726	*RGS17-OPRM1*	A	0.42	0.26–0.67	2.5 × 10^−4^	dominant
19	rs1671152	*GP6*	A	2.24	1.42–3.53	5.2 × 10^−4^	dominant
6	rs7454156	*BMP6*	G	5.14	2.54–10.37	4.87 × 10^−6^	recessive

Logistic regression assuming a dominant genetic model adjusted by age, sex, principal components, and median diastolic blood pressure. Chromosome (CHR), SNP ID (rs number), gene, coded allele (CA), odds ratio (OR), 95% confidence interval (CI), *p*-value, and assumed genetic model are given

While certain variants were found to be associated with POAG in both dominant/additive and recessive/additive models, we found that no SNPs were consistently associated with POAG in both dominant and recessive models.

## Discussion

Epidemiologic and clinical studies have demonstrated that POAG risk is higher in African-descent populations compared with other populations such as European Americans. To identify genetic variants associated with POAG risk that are specific to African-descent populations or shared across world populations, we identified African American POAG cases and controls in a clinic setting using electronic health records to conduct genetic associations studies in the fine-mapped region of *CDKN2B-AS1* and Metabochip-wide [34]. Overall, we found evidence that the percentage of African ancestry at *CDKN2B-AS1* was strongly correlated with POAG case status (*p* = 2 × 10^− 6^). POAG cases on average contained 90% African ancestral alleles at the *CDKN2B-AS1* region versus controls which were only 58% African, suggesting that African-specific variation may indeed being driving risk at this locus. Additionally, the lack of strong statistical associations with individual SNPs but an association with gene-based African ancestry suggests that gene x gene or gene x environment interactions may be involved but which will require larger sample sizes to accurately assess the possibility.

Common variants in *CDKN2B-ASI* are consistently associated with POAG in European-descent populations [25, 27, 51]. We [34] and others [27, 52, 53] have demonstrated that these same variants are inconsistently associated with POAG in African-descent populations. The lack of association in African-descent populations is likely due to limited power, a consequence of smaller sample sizes and considerably lower allele frequencies compared with studies of European-descent populations. For example, *CDKN2B-AS1* rs2157719 has a minor allele frequency of 3 and 46% in African Americans (ASW) and Europeans (CEU), respectively, in phase 3 of the 1000 Genomes Project. Originally discovered in a European POAG cohort [25], rs2157719 was found to be associated with optic nerve degeneration in glaucoma patients with an odds ratio of 1.45. The present study is powered (80%) to detect associations for common variants (≥15% MAF) with large effect sizes (at least 2.9 odds ratio) at genome-wide significance (5 × 10^− 8^). The small sample size of this study is underpowered to detect associations for less frequent variants and/or variants with smaller effect sizes. Although we could not generalize these associations in this study sample, we note that this locus is still important in POAG risk as evidenced by the association between African ancestry at this locus and POAG. It is also interesting to note that the *CDKN2B-AS1* rs2157719 allele associated with lower odds of POAG (C/G) is the ancestral allele yet the minor allele in all 1000 Genomes Project populations. The high frequency of the derived allele at rs2157719 may be due to chance, positive selection (and possible antagonistic pleiotropy), or an error in ancestral allele assignment, among other possibilities.

## Conclusions

Here, we show a significant association between POAG risk and local African genetic ancestry at *CDKN2B-AS1* (*p* = 2 × 10^− 6^). While not identifying significant single SNP-POAG associations after adjusting for multiple testing, the results still suggest that *CDKN2B-AS1* is an important locus of POAG risk among African Americans, warranting further investigation to identify genetic variants or epigenetic regulators that may be acting in conjunction with this locus. When gauging the strengths and limitations of this study, perhaps its greatest strength is the expansion of knowledge in African Americans, a population far too often underrepresented in biomedical research [58]. Additional strengths involve the utilization of electronic health records as a cost efficient and data-dense resource for studies. A major limitation of our study is statistical power. Nevertheless, this study is one of only a handful to assess the genetic architecture of POAG in African Americans.

## Additional file

Additional file 1: **Table S1.** Genome-wide association study (GWAS)-identified index variants associated with ocular disease and related traits directly assayed by the Illumina Metabochip. **Table S2.** The 100 most significant results for the genetic association analysis of the Metabochip and African American primary open-angle glaucoma cases (*n* = 138) and controls (*n* = 1376). **Table S3.** Results (*p* < 0.0001) for RECESSIVE genetic models for Metabochip-wide tests of association in African American primary open-angle glaucoma cases (*n* = 138) and controls (*n* = 1376). **Table S4.** Results (*p* < 0.0001) for DOMINANT genetic models for Metabochip-wide tests of association in African American primary open-angle glaucoma cases (*n* = 138) and controls (*n* = 1376). (DOCX 52 kb)

## Abbreviations

EAGLE: Epidemiologic Architecture for Genes Linked to Environment
GWAS: Genome-wide association studies
HDL-C: High density lipoprotein cholesterol
IRB: Vanderbilt Institutional Review Board
LAMP: Local Ancestry in admixed Populations
MAF: Minor allele frequency
OR: Odds ratio
PAGE: Population Architecture using Genomics and Epidemiology
PC: Principal components
POAG: Primary open-angle glaucoma
VUMC: Vanderbilt University Medical Center
