# Supplementary material for: Local genetic ancestry in CDKN2B-AS1 is associated with primary open-angle glaucoma in an African American cohort extracted from de-identified electronic health records
Source: BMC Med Genomics. 2018 Sep 14;11(Suppl 3):70. doi: 10.1186/s12920-018-0392-4 (PMC6157155; doi:10.1186/s12920-018-0392-4)
Supplement: Supplementary file 1 — Table S1. Genome-wide association study (GWAS)-identified index variants associated with ocular disease and related traits directly assayed by the Illumina Metabochip. Table S2. The 100 most significant results for the genetic association analysis of the Metabochip and African American primary open-angle glaucoma cases (n = 138) and controls (n = 1376). Table S3. Results (p < 0.0001) for RECESSIVE genetic models for Metabochip-wide tests of association in African American primary open-angle glaucoma cases (n = 138) and controls (n = 1376). Table S4. Results (p < 0.0001) for DOMINANT genetic models for Metabochip-wide tests of association in African American primary open-angle glaucoma cases (n = 138) and controls (n = 1376). (DOCX 52 kb) [file 12920_2018_392_MOESM1_ESM.docx]

**Local genetic ancestry in *CDKN2B-AS1* is associated with primary open-angle glaucoma in an African American cohort extracted from de-identified electronic health records**

Nicole A. Restrepo^1^, Sarah M. Laper^2^, Eric Farber-Eger^3^, Dana C. Crawford^1*^

^1^ Department of Population and Quantitative Health Sciences, Institute for Computational Biology, Case Western Reserve University, Cleveland, OH, USA

^2^Eastern Virginia Medical School, Norfolk, VA, USA

^3^Vanderbilt Institute for Clinical and Translational Research, Vanderbilt University Medical Center, Nashville, TN, USA

*Corresponding author

Dana Crawford, PhD

2103 Cornell Road

Wolstein Research Building, Suite 2-527

Cleveland, OH 44106

(216) 368-5546

[dana.crawford@case.edu](mailto:dana.crawford@case.edu)

**Table S1. Genome-wide association study (GWAS)-identified index variants associated with ocular disease and related traits directly assayed by the Illumina Metabochip.** Variants listed by chromosome:position are based on the original Metabochip design (on build 36). Abbreviations: age-related macular degeneration (AMD), cup-to-disc (CTD), glycated hemoglobin (HbA1c), intraocular pressure (IOP), normal pressure glaucoma (NPG), normal tension (NT), primary open-angle glaucoma (POAG), PubMed ID (PMID)

| **SNP** | **CHR** | **Closest Gene** | **Disease or Trait** | **PMID** |
| --- | --- | --- | --- | --- |
| **rs994767** | 1 | *ZC3H11B* | Ocular axial length | 24144296 |
| **rs11755724** | 6 | *RREB1* | AMD | 20385826 |
| **rs2070600** | 6 | *AGER* | Diabetic retinopathy | 21067572 |
| **rs730497** | 7 | *GCK* | HbA1c | 19096518 |
| **rs10237118** | 7 | *TRIM24* | Optic disc size (cup) | 20395239 |
| **rs13266634** | 8 | *EIF3H* | HbA1c | 19734900 |
| **rs564398** | 9 | *CDKN2B* | POAG | 22428042 |
| **rs523096** | 9 | *CDKN2B* | NT-Glaucoma | 22792221 |
| **rs3217992** | 9 | *CDKN2B* | NPG | 22570617 |
| **rs2157719** | 9 | *CDKN2B* | POAG | 22570617 |
| **rs1412829** | 9 | *CDKN2B* | NPG | 22570617 |
| **rs1063192** | 9 | *CDKN2B* | POAG | 22419738 |
| **rs1063192** | 9 | *CDKN2B* | POAG and CTD ratio | 22419738  22570617 |
| **rs7894966** | 10 | GAS7 | IOP | 22570627 |
| **rs7072268** | 10 | *HK1* | HbA1c | 19096518 |
| **rs3858145** | 10 | *ATOH7* | Optic disc size (cup) | 20395239 |
| **rs3793917** | 10 | *ARMS2* | AMD | 20385819 |
| **rs10490924** | 10 | *ARMS2* | AMD | 20385826 |
| **rs11812882** | 10 | *CISD1* | Diabetic retinopathy | 20871662 |
| **rs5742629** | 12 | *IGF1* | Myopia (extreme) | 22509095 |
| **rs10858945** | 12 | *LOC338758* | Optic disc size (cup) | 20395239 |
| **rs10483727** | 14 | *AKR1B1P5* | POAG and Optic disc size (rim) | 20548946  22419738  22570617 |
| **rs493258** | 15 | *RPL28P4* | AMD | 20385819 |
| **rs10468017** | 15 | *LIPC* | AMD | 20385826 |
| **rs3764261** | 16 | *CETP* | AMD | 20385819 |
| **rs10521145** | 16 | *CCDC101* | Diabetic retinopathy | 22427569 |
| **rs151227** | 16 | *NUPR1* | Diabetic retinopathy | 21441570 |
| **rs151230** | 16 | *CCDC101* | Diabetic retinopathy | 21441570 |
| **rs151229** | 16 | *CCDC101* | Diabetic retinopathy | 21441570 |
| **rs10521145** | 16 | *CCDC101* | Diabetic retinopathy | 21441570 |
| **rs11641853** | 16 | *CCDC101* | Diabetic retinopathy | 21441570 |
| **rs11074904** | 16 | *SULT1A1* | Diabetic retinopathy | 21441570 |
| **rs1109739** | 16 | *16q12* | POAG | 22661486 |
| **rs134173** | 22 | *CHEK2* | CTD ratio | 22570617 |

**Table S2.** **The one hundred most significant results for the genetic association analysis of the Metabochip and African Americans POAG cases (n=138) and controls (n=1,376).** Logistic regression assuming an additive genetic model was performed for adjusted for age, sex, principal components, and median diastolic blood pressure. Variants listed by chromosome:position are based on the original Metabochip design (on build 36).

| CHR | SNP | Coded Allele | OR | 95% CI-L | 95% CI-U | | P-value |
| --- | --- | --- | --- | --- | --- | --- | --- |
| 1 | chr1:228347779 | A | 2.373 | 1.563 | 3.603 | 5.00E-05 | |
| 1 | chr1:228354829 | C | 2.094 | 1.447 | 3.028 | 8.73E-05 | |
| 21 | rs9982695 | A | 2.09 | 1.446 | 3.02 | 8.74E-05 | |
| 6 | chr6:25793471 | C | 1.943 | 1.392 | 2.714 | 9.64E-05 | |
| 4 | rs3775202 | G | 1.921 | 1.383 | 2.666 | 9.70E-05 | |
| 2 | rs13423742 | C | 3.048 | 1.73 | 5.369 | 0.000115 | |
| 6 | rs7454156 | G | 2.078 | 1.427 | 3.026 | 0.000138 | |
| 6 | rs9479726 | A | 0.405 | 0.2536 | 0.6469 | 0.000155 | |
| 19 | rs1671152 | A | 1.916 | 1.367 | 2.686 | 0.000161 | |
| 10 | rs286489 | A | 1.901 | 1.358 | 2.66 | 0.00018 | |
| 5 | rs4336354 | G | 2.511 | 1.549 | 4.07 | 0.000187 | |
| 16 | rs7190904 | A | 2.293 | 1.483 | 3.546 | 0.000192 | |
| 16 | chr16:52507417 | A | 2.022 | 1.395 | 2.931 | 0.000202 | |
| 5 | rs7714384 | A | 1.858 | 1.336 | 2.584 | 0.000232 | |
| 7 | chr7:14708236 | G | 0.3397 | 0.1908 | 0.6047 | 0.000243 | |
| 16 | rs1424077 | G | 2.259 | 1.461 | 3.492 | 0.000246 | |
| 6 | rs6929849 | A | 0.5075 | 0.3532 | 0.7293 | 0.000246 | |
| 4 | rs17028407 | A | 2.735 | 1.596 | 4.688 | 0.000252 | |
| 4 | chr4:88283455 | G | 1.876 | 1.338 | 2.63 | 0.000266 | |
| 19 | rs2910368 | G | 2.299 | 1.468 | 3.602 | 0.000276 | |
| 4 | chr4:88259933 | A | 1.875 | 1.336 | 2.631 | 0.000279 | |
| 13 | rs1547918 | G | 2.302 | 1.468 | 3.609 | 0.00028 | |
| 1 | rs649214 | G | 1.848 | 1.326 | 2.577 | 0.000291 | |
| 13 | chr13:109680753 | A | 2.368 | 1.482 | 3.784 | 0.000314 | |
| 16 | chr16:52509162 | A | 2.085 | 1.398 | 3.111 | 0.000317 | |
| 5 | rs17066506 | A | 2.118 | 1.408 | 3.187 | 0.000319 | |
| 4 | rs6832117 | A | 1.809 | 1.306 | 2.505 | 0.00036 | |
| 8 | rs10956525 | G | 3.112 | 1.667 | 5.813 | 0.000368 | |
| 1 | chr1:109506916 | G | 2.062 | 1.384 | 3.072 | 0.000377 | |
| 4 | rs1408 | G | 1.854 | 1.317 | 2.611 | 0.000409 | |
| 8 | rs6415517 | A | 2.693 | 1.554 | 4.664 | 0.000411 | |
| 4 | chr4:88242323 | G | 1.915 | 1.335 | 2.747 | 0.000415 | |
| 5 | chr5:157782818 | A | 2.403 | 1.477 | 3.91 | 0.000418 | |
| 3 | rs4647226 | A | 3.01 | 1.629 | 5.559 | 0.000433 | |
| 2 | rs11127229 | A | 2.118 | 1.394 | 3.218 | 0.000439 | |
| 4 | chr4:88259209 | A | 1.85 | 1.313 | 2.607 | 0.00044 | |
| 2 | rs3768641 | G | 2.022 | 1.362 | 3 | 0.000472 | |
| 13 | rs449674 | A | 0.3867 | 0.227 | 0.6587 | 0.000473 | |
| 22 | rs9608416 | A | 2.001 | 1.355 | 2.953 | 0.000483 | |
| 4 | chr4:88272109 | G | 1.834 | 1.304 | 2.579 | 0.000491 | |
| 6 | rs241407 | A | 2.215 | 1.416 | 3.465 | 0.000494 | |
| 8 | rs2158588 | A | 2.755 | 1.552 | 4.891 | 0.000536 | |
| 19 | chr19:19449565 | A | 2.478 | 1.478 | 4.154 | 0.000579 | |
| 15 | rs11639241 | C | 1.815 | 1.29 | 2.553 | 0.000614 | |
| 7 | chr7:14720840 | A | 0.3619 | 0.2021 | 0.6479 | 0.000625 | |
| 5 | rs1529707 | A | 0.5403 | 0.3796 | 0.769 | 0.00063 | |
| 13 | rs1322379 | G | 1.902 | 1.315 | 2.75 | 0.000634 | |
| 11 | chr11:10305291 | G | 2.244 | 1.411 | 3.568 | 0.000635 | |
| 2 | rs1357011 | C | 2.641 | 1.51 | 4.617 | 0.000658 | |
| 19 | rs2967732 | C | 1.99 | 1.339 | 2.959 | 0.000669 | |
| 5 | rs10063054 | C | 1.758 | 1.27 | 2.434 | 0.00068 | |
| 1 | rs2422286 | A | 1.93 | 1.32 | 2.82 | 0.000688 | |
| 6 | rs12215670 | G | 2.639 | 1.505 | 4.628 | 0.000706 | |
| 5 | rs9885411 | G | 2.933 | 1.573 | 5.472 | 0.000716 | |
| 7 | rs4720833 | A | 0.4503 | 0.2836 | 0.715 | 0.000721 | |
| 3 | rs4678836 | A | 1.872 | 1.301 | 2.693 | 0.00073 | |
| 5 | chr5:157674339 | A | 1.777 | 1.273 | 2.481 | 0.000737 | |
| 6 | chr6:25818742 | C | 1.773 | 1.271 | 2.474 | 0.000748 | |
| 6 | rs3869129 | A | 0.5399 | 0.377 | 0.7733 | 0.000773 | |
| 19 | chr19:19468633 | G | 2.423 | 1.446 | 4.061 | 0.00078 | |
| 2 | rs3097385 | G | 2.002 | 1.335 | 3.003 | 0.000792 | |
| 16 | rs237174 | A | 1.925 | 1.313 | 2.822 | 0.000793 | |
| 6 | rs10456759 | C | 1.83 | 1.284 | 2.607 | 0.000823 | |
| 10 | rs11196187 | A | 2.694 | 1.506 | 4.819 | 0.000838 | |
| 5 | chr5:157668311 | G | 1.757 | 1.261 | 2.448 | 0.000874 | |
| 4 | rs223482 | A | 1.748 | 1.258 | 2.429 | 0.000886 | |
| 13 | rs3924002 | G | 2.137 | 1.365 | 3.346 | 0.000897 | |
| 11 | chr11:2876684 | A | 1.994 | 1.327 | 2.998 | 0.000902 | |
| 17 | rs2659015 | A | 2.046 | 1.341 | 3.124 | 0.000908 | |
| 14 | rs10135856 | A | 0.5781 | 0.4181 | 0.7993 | 0.000917 | |
| 5 | chr5:157767459 | A | 0.5537 | 0.3903 | 0.7857 | 0.000928 | |
| 13 | rs17591848 | G | 1.908 | 1.302 | 2.797 | 0.000929 | |
| 5 | chr5:157760095 | G | 0.5543 | 0.3907 | 0.7864 | 0.000945 | |
| 5 | chr5:157766571 | G | 0.5543 | 0.3907 | 0.7864 | 0.000945 | |
| 5 | chr5:157758859 | A | 0.5545 | 0.3909 | 0.7866 | 0.000948 | |
| 15 | chr15:73056964 | A | 2.521 | 1.457 | 4.362 | 0.00095 | |
| 5 | chr5:157758831 | A | 0.5554 | 0.3917 | 0.7877 | 0.000972 | |
| 12 | chr12:48529939 | G | 1.748 | 1.254 | 2.436 | 0.000975 | |
| 9 | rs7863513 | A | 0.54 | 0.3744 | 0.7789 | 0.000977 | |
| 10 | rs4347309 | G | 1.792 | 1.266 | 2.536 | 0.000989 | |
| 6 | chr6:25818066 | G | 0.5682 | 0.4058 | 0.7957 | 0.001001 | |
| 8 | rs11863 | A | 1.767 | 1.258 | 2.48 | 0.001009 | |
| 5 | rs4700135 | G | 0.5332 | 0.3661 | 0.7765 | 0.001043 | |
| 10 | rs853928 | A | 0.4842 | 0.3138 | 0.7472 | 0.001049 | |
| 5 | chr5:157736208 | A | 0.5515 | 0.386 | 0.7879 | 0.001077 | |
| 5 | chr5:157759964 | A | 0.5573 | 0.3925 | 0.7913 | 0.001079 | |
| 1 | rs11810369 | A | 1.726 | 1.244 | 2.395 | 0.001081 | |
| 6 | rs9479660 | G | 1.752 | 1.252 | 2.453 | 0.001087 | |
| 18 | rs1786153 | A | 1.723 | 1.243 | 2.389 | 0.001103 | |
| 17 | rs16956560 | G | 1.754 | 1.251 | 2.457 | 0.001104 | |
| 5 | chr5:157764878 | G | 0.5579 | 0.3928 | 0.7924 | 0.001115 | |
| 6 | rs3201892 | C | 0.5271 | 0.3585 | 0.7749 | 0.001126 | |
| 2 | rs13394146 | A | 1.738 | 1.246 | 2.425 | 0.001141 | |
| 2 | rs4849816 | A | 1.734 | 1.245 | 2.417 | 0.001145 | |
| 6 | rs6455482 | G | 1.851 | 1.277 | 2.682 | 0.001149 | |
| 2 | rs12613548 | A | 0.4604 | 0.2884 | 0.7351 | 0.001158 | |
| 1 | rs2039988 | A | 2.045 | 1.327 | 3.15 | 0.001176 | |
| 12 | chr12:48488687 | A | 1.76 | 1.251 | 2.476 | 0.001182 | |
| 6 | chr6:25833213 | A | 0.5745 | 0.4109 | 0.8031 | 0.001185 | |
| 8 | rs11784268 | C | 1.871 | 1.281 | 2.734 | 0.001203 | |
| OR = odds ratio  CI-L = lower confidence interval  CI-U = upper confidence interval | | | | | | | |

**Table S3. Results (p < 0.0001) for RECESSIVE genetic models for Metabochip-wide tests of association in African Americans POAG cases (n=138) and controls (n=1,376).** Logistic regression assuming a recessive genetic model was performed adjusted for age, sex, principal components, and median diastolic blood pressure. Variants listed by chromosome:position are based on the original Metabochip design (on build 36).

| CHR | SNP | Coded Allele | TEST | OR | CI- L95 | CI-U95 | P-value |
| --- | --- | --- | --- | --- | --- | --- | --- |
| 2 | rs10180522 | C | REC | 6.286 | 2.93 | 13.49 | 2.35E-06 |
| 12 | rs2520497 | G | REC | 5.487 | 2.666 | 11.3 | 3.81E-06 |
| 6 | rs7454156 | G | REC | 5.141 | 2.547 | 10.37 | 4.87E-06 |
| 8 | rs10102164 | A | REC | 7.176 | 3.049 | 16.89 | 6.39E-06 |
| 12 | chr12:64513660 | A | REC | 6.25 | 2.755 | 14.18 | 1.17E-05 |
| 12 | chr12:64509572 | C | REC | 6.201 | 2.742 | 14.03 | 1.18E-05 |
| 12 | chr12:64509813 | G | REC | 6.197 | 2.74 | 14.02 | 1.19E-05 |
| 8 | rs10104997 | A | REC | 6.143 | 2.67 | 14.13 | 1.96E-05 |
| 12 | chr12:64518645 | G | REC | 5.873 | 2.601 | 13.26 | 2.05E-05 |
| 5 | chr5:156301749 | G | REC | 5.651 | 2.541 | 12.57 | 2.17E-05 |
| 4 | hg18_4_6657823 | A | REC | 7.438 | 2.918 | 18.96 | 2.64E-05 |
| 12 | chr12:64499763 | T | REC | 5.585 | 2.492 | 12.52 | 2.95E-05 |
| 13 | rs9593772 | G | REC | 7.927 | 2.999 | 20.95 | 2.98E-05 |
| 5 | chr5:157674339 | A | REC | 3.245 | 1.865 | 5.647 | 3.13E-05 |
| 5 | chr5:157668311 | G | REC | 3.122 | 1.821 | 5.352 | 3.50E-05 |
| 1 | chr1:74765315 | A | REC | 15.76 | 4.241 | 58.57 | 3.84E-05 |
| 2 | rs11127229 | A | REC | 6.677 | 2.636 | 16.91 | 6.22E-05 |
| 4 | rs4608786 | A | REC | 2.728 | 1.667 | 4.463 | 6.48E-05 |
| 1 | chr1:56792432 | G | REC | 4.574 | 2.136 | 9.794 | 9.10E-05 |
| 20 | rs1555318 | A | REC | 7.425 | 2.713 | 20.32 | 9.49E-05 |
| 2 | chr2:21143572 | A | REC | 2.792 | 1.666 | 4.679 | 9.66E-05 |
| 2 | chr2:21144817 | G | REC | 2.792 | 1.666 | 4.679 | 9.66E-05 |
| 5 | rs10061608 | A | REC | 30.61 | 5.411 | 173.2 | 0.000109 |
| 4 | chr4:88259933 | A | REC | 3.14 | 1.756 | 5.612 | 0.000113 |
| 16 | rs16949396 | C | REC | 3.12 | 1.748 | 5.568 | 0.000118 |
| 8 | rs9298506 | G | REC | 5.616 | 2.332 | 13.52 | 0.000119 |
| 19 | rs8111710 | A | REC | 4.723 | 2.137 | 10.44 | 0.000124 |
| 20 | rs2425785 | C | REC | 2.318 | 1.502 | 3.578 | 0.000146 |
| 11 | chr11:2692389 | G | REC | 13.6 | 3.521 | 52.56 | 0.000154 |
| 5 | rs13178142 | A | REC | 7.94 | 2.714 | 23.23 | 0.000155 |
| 2 | chr2:21157121 | A | REC | 2.463 | 1.538 | 3.944 | 0.000175 |
| 15 | rs11639241 | C | REC | 3.239 | 1.752 | 5.989 | 0.000178 |
| 6 | rs12175489 | A | REC | 16.74 | 3.827 | 73.24 | 0.000182 |
| 3 | rs9814870 | G | REC | 5.539 | 2.244 | 13.67 | 0.000205 |
| 19 | rs16968492 | G | REC | 4.031 | 1.927 | 8.431 | 0.000213 |
| 3 | rs1401970 | A | REC | 7.636 | 2.59 | 22.52 | 0.000229 |
| 11 | chr11:47334972 | A | REC | 2.956 | 1.66 | 5.264 | 0.000232 |
| 18 | rs1457489 | A | REC | 2.437 | 1.516 | 3.917 | 0.000235 |
| 20 | rs6140807 | A | REC | 41.45 | 5.69 | 301.9 | 0.000237 |
| 4 | rs2725221 | G | REC | 7.175 | 2.486 | 20.71 | 0.000269 |
| 11 | chr11:47367464 | A | REC | 2.655 | 1.569 | 4.493 | 0.000275 |
| 8 | rs7821465 | G | REC | 2.606 | 1.551 | 4.378 | 0.000297 |
| 8 | rs9886613 | G | REC | 11.54 | 3.062 | 43.49 | 0.000303 |
| 8 | rs10088001 | A | REC | 11.54 | 3.062 | 43.49 | 0.000303 |
| 13 | chr13:109640159 | A | REC | 2.199 | 1.434 | 3.373 | 0.000303 |
| 4 | rs1803037 | A | REC | 11.61 | 3.068 | 43.95 | 0.000305 |
| 6 | chr6:6689577 | G | REC | 22.49 | 4.121 | 122.7 | 0.000324 |
| 2 | rs6434787 | A | REC | 2.594 | 1.54 | 4.37 | 0.000342 |
| 2 | rs668948 | A | REC | 2.338 | 1.469 | 3.722 | 0.000343 |
| 1 | chr1:74763069 | A | REC | 5.863 | 2.224 | 15.46 | 0.000348 |
| 4 | rs6832117 | A | REC | 2.331 | 1.466 | 3.707 | 0.00035 |
| 7 | chr7:71711652 | A | REC | 7.254 | 2.442 | 21.55 | 0.000362 |
| 17 | chr17:40552542 | A | REC | 13.94 | 3.274 | 59.38 | 0.000365 |
| 17 | chr17:40552649 | C | REC | 13.94 | 3.274 | 59.38 | 0.000365 |
| 7 | chr7:71712086 | G | REC | 7.233 | 2.434 | 21.5 | 0.00037 |
| 1 | chr1:74759256 | G | REC | 6.988 | 2.396 | 20.38 | 0.00037 |
| 1 | chr1:74760492 | T | REC | 6.988 | 2.396 | 20.38 | 0.00037 |
| 2 | rs541041 | A | REC | 2.328 | 1.462 | 3.707 | 0.00037 |
| 17 | chr17:40554561 | A | REC | 15.68 | 3.434 | 71.55 | 0.000381 |
| 7 | chr7:71816784 | G | REC | 7.16 | 2.417 | 21.21 | 0.000382 |
| 10 | chr10:104483434 | A | REC | 14.05 | 3.27 | 60.4 | 0.000382 |
| 16 | rs237174 | A | REC | 4.145 | 1.891 | 9.085 | 0.000383 |
| 6 | rs10946405 | A | REC | 3.663 | 1.789 | 7.501 | 0.000384 |
| 7 | rs804025 | G | REC | 3.647 | 1.786 | 7.45 | 0.000384 |
| 4 | rs16844364 | A | REC | 5.055 | 2.065 | 12.37 | 0.000388 |
| 10 | rs11017721 | A | REC | 4.248 | 1.91 | 9.448 | 0.000389 |
| 2 | rs1367117 | A | REC | 7.415 | 2.44 | 22.53 | 0.000411 |
| 17 | rs16960077 | G | REC | 39.77 | 5.132 | 308.2 | 0.000423 |
| 16 | chr16:52685184 | A | REC | 37.32 | 4.972 | 280.1 | 0.000432 |
| 9 | chr9:106686045 | A | REC | 34.06 | 4.769 | 243.2 | 0.000436 |
| 5 | chr5:156351368 | A | REC | 17.98 | 3.593 | 90.01 | 0.000437 |
| 12 | rs1946298 | G | REC | 3.147 | 1.661 | 5.965 | 0.00044 |
| 8 | rs6991922 | A | REC | 2.36 | 1.462 | 3.811 | 0.000443 |
| 16 | chr16:52546608 | C | REC | 7.466 | 2.427 | 22.96 | 0.000454 |
| 15 | rs3848109 | A | REC | 4.152 | 1.872 | 9.208 | 0.000461 |
| 15 | rs1343843 | A | REC | 4.152 | 1.872 | 9.208 | 0.000461 |
| 2 | chr2:226723504 | G | REC | 4.023 | 1.844 | 8.775 | 0.00047 |
| 2 | chr2:226726050 | G | REC | 4.023 | 1.844 | 8.775 | 0.00047 |
| 5 | rs17067254 | A | REC | 4.212 | 1.881 | 9.43 | 0.000471 |
| 5 | rs16896181 | G | REC | 55.44 | 5.818 | 528.2 | 0.000481 |
| 13 | chr13:109640154 | A | REC | 2.137 | 1.393 | 3.278 | 0.000501 |
| 1 | rs11576201 | A | REC | 14.45 | 3.209 | 65.02 | 0.000503 |
| 5 | rs2455249 | G | REC | 9.135 | 2.617 | 31.89 | 0.000524 |
| 3 | rs6440196 | A | REC | 6.187 | 2.208 | 17.34 | 0.000526 |
| 5 | chr5:156334111 | G | REC | 15.26 | 3.267 | 71.28 | 0.000529 |
| 8 | rs13439262 | G | REC | 9.803 | 2.694 | 35.67 | 0.000533 |
| 6 | rs2803191 | A | REC | 2.235 | 1.418 | 3.524 | 0.000533 |
| 4 | chr4:88272109 | G | REC | 2.868 | 1.58 | 5.207 | 0.000536 |
| 1 | rs12131641 | A | REC | 33.61 | 4.594 | 245.9 | 0.000537 |
| 11 | rs10500666 | A | REC | 24.49 | 3.996 | 150.1 | 0.000545 |
| 14 | rs943997 | A | REC | 5.616 | 2.105 | 14.98 | 0.000568 |
| 2 | chr2:705029 | G | REC | 3.529 | 1.719 | 7.245 | 0.000591 |
| 6 | chr6:118648180 | A | REC | 5.728 | 2.115 | 15.52 | 0.000597 |
| 18 | rs1786153 | A | REC | 2.168 | 1.394 | 3.373 | 0.000599 |
| 22 | rs130743 | A | REC | 5.443 | 2.064 | 14.35 | 0.000614 |
| 5 | rs10069 | A | REC | 4.864 | 1.967 | 12.03 | 0.000617 |
| 1 | chr1:176069786 | A | REC | 5.226 | 2.023 | 13.5 | 0.000637 |
| 6 | rs369150 | A | REC | 6.252 | 2.184 | 17.9 | 0.000638 |
| 7 | rs7780323 | C | REC | 2.516 | 1.481 | 4.272 | 0.00064 |
| 2 | rs2045245 | A | REC | 3.744 | 1.752 | 8.001 | 0.000656 |
| 10 | rs12242535 | A | REC | 5.269 | 2.02 | 13.74 | 0.00068 |
| 4 | rs1408 | G | REC | 2.812 | 1.548 | 5.108 | 0.000687 |
| 1 | chr1:74775137 | T | REC | 6.266 | 2.169 | 18.1 | 0.000698 |
| 4 | rs7678362 | G | REC | 2.86 | 1.556 | 5.259 | 0.000717 |
| 12 | rs2806311 | G | REC | 2.209 | 1.395 | 3.499 | 0.000727 |
| 16 | rs12103094 | C | REC | 8.152 | 2.398 | 27.71 | 0.000776 |
| 12 | chr12:64533064 | G | REC | 3.321 | 1.649 | 6.687 | 0.000777 |
| 4 | rs10014069 | A | REC | 34.13 | 4.348 | 267.8 | 0.000785 |
| 16 | chr16:52499785 | G | REC | 3.443 | 1.673 | 7.084 | 0.000786 |
| 1 | chr1:160488903 | G | REC | 9.104 | 2.506 | 33.07 | 0.000791 |
| 12 | rs11830407 | A | REC | 4.05 | 1.786 | 9.183 | 0.00081 |
| 3 | chr3:139442347 | G | REC | 29.6 | 4.076 | 214.9 | 0.000811 |
| 20 | chr20:38554907 | C | REC | 35.43 | 4.389 | 286 | 0.000814 |
| 1 | rs12089328 | A | REC | 30.68 | 4.123 | 228.4 | 0.000829 |
| 3 | rs9858613 | A | REC | 2.754 | 1.52 | 4.99 | 0.000836 |
| 7 | chr7:14635830 | G | REC | 2.215 | 1.389 | 3.533 | 0.000838 |
| 5 | rs7714384 | A | REC | 2.129 | 1.366 | 3.319 | 0.000848 |
| 11 | rs12785289 | G | REC | 17.02 | 3.207 | 90.32 | 0.000874 |
| 15 | rs990579 | G | REC | 5.091 | 1.95 | 13.29 | 0.000888 |
| 7 | rs1012036 | A | REC | 12.16 | 2.787 | 53.09 | 0.00089 |
| 7 | chr7:14641422 | C | REC | 2.2 | 1.381 | 3.505 | 0.000911 |
| 7 | chr7:14637666 | A | REC | 2.201 | 1.381 | 3.508 | 0.000912 |
| 3 | chr3:38662653 | A | REC | 3.257 | 1.62 | 6.551 | 0.000926 |
| 10 | rs7088114 | G | REC | 2.183 | 1.375 | 3.464 | 0.000927 |
| 2 | chr2:21169169 | A | REC | 2.222 | 1.385 | 3.566 | 0.000934 |
| 7 | chr7:14642018 | A | REC | 2.194 | 1.377 | 3.496 | 0.000946 |
| 16 | rs4985124 | C | REC | 3.226 | 1.611 | 6.461 | 0.000948 |
| 13 | rs1781576 | A | REC | 41.31 | 4.54 | 375.8 | 0.000957 |
| 1 | rs11810751 | C | REC | 2.675 | 1.491 | 4.8 | 0.000972 |
| 2 | rs16865722 | A | REC | 7.826 | 2.304 | 26.59 | 0.000975 |
| 8 | rs4144757 | A | REC | 2.721 | 1.499 | 4.939 | 0.000997 |

**Table S4. Results (p < 0.0001) for DOMINANT genetic models for Metabochip-wide tests of association in African Americans POAG cases (n=138) and controls (n=1,376).** Logistic regression assuming a dominant genetic model was performed adjusted for age, sex, principal components, and median diastolic blood pressure. Variants listed by chromosome:position are based on the original Metabochip design (on build 36).

| CHR |  | SNP | A1 | TEST | OR | L95 | U95 | P |
| --- | --- | --- | --- | --- | --- | --- | --- | --- |
| 13 |  | chr13:109806392 | A | DOM | 2.721 | 1.68 | 4.406 | 4.74E-05 |
| 1 |  | chr1:228347779 | A | DOM | 2.425 | 1.569 | 3.746 | 6.59E-05 |
| 5 |  | rs10063054 | C | DOM | 2.944 | 1.729 | 5.011 | 6.97E-05 |
| 16 |  | rs1424077 | G | DOM | 2.437 | 1.56 | 3.808 | 9.10E-05 |
| 4 |  | rs1605923 | C | DOM | 2.46 | 1.558 | 3.886 | 0.000113 |
| 3 |  | rs7426919 | A | DOM | 0.4404 | 0.2902 | 0.6685 | 0.000117 |
| 6 |  | rs12215670 | G | DOM | 2.936 | 1.686 | 5.111 | 0.000141 |
| 1 |  | chr1:62687120 | A | DOM | 2.334 | 1.508 | 3.612 | 0.000142 |
| 13 |  | rs449674 | A | DOM | 0.3708 | 0.222 | 0.6194 | 0.000151 |
| 11 |  | chr11:10275899 | A | DOM | 2.522 | 1.562 | 4.073 | 0.000155 |
| 1 |  | chr1:228354829 | C | DOM | 2.235 | 1.472 | 3.394 | 0.000161 |
| 11 |  | chr11:10305291 | G | DOM | 2.525 | 1.56 | 4.086 | 0.000162 |
| 11 |  | chr11:10276738 | A | DOM | 2.511 | 1.555 | 4.054 | 0.000166 |
| 4 |  | rs7660298 | A | DOM | 2.25 | 1.475 | 3.434 | 0.000168 |
| 1 |  | rs380149 | G | DOM | 2.287 | 1.486 | 3.519 | 0.000169 |
| 3 |  | rs812657 | A | DOM | 2.265 | 1.479 | 3.47 | 0.00017 |
| 11 |  | chr11:10280435 | C | DOM | 2.497 | 1.547 | 4.031 | 0.00018 |
| 11 |  | chr11:10279520 | T | DOM | 2.495 | 1.546 | 4.028 | 0.000182 |
| 11 |  | chr11:48000677 | A | DOM | 2.725 | 1.61 | 4.61 | 0.000187 |
| 13 |  | chr13:109811204 | A | DOM | 2.604 | 1.575 | 4.303 | 0.000189 |
| 3 |  | chr3:86073380 | A | DOM | 2.232 | 1.464 | 3.403 | 0.00019 |
| 2 |  | rs13423742 | C | DOM | 2.816 | 1.631 | 4.862 | 0.000202 |
| 3 |  | rs793445 | A | DOM | 2.242 | 1.464 | 3.433 | 0.000205 |
| 3 |  | chr3:86063771 | G | DOM | 2.216 | 1.454 | 3.379 | 0.000217 |
| 11 |  | chr11:10273292 | G | DOM | 2.461 | 1.525 | 3.971 | 0.000225 |
| 6 |  | rs4708849 | A | DOM | 0.4411 | 0.2852 | 0.6822 | 0.000234 |
| 3 |  | chr3:86098949 | A | DOM | 2.197 | 1.442 | 3.349 | 0.000251 |
| 6 |  | rs9479726 | A | DOM | 0.4213 | 0.2651 | 0.6695 | 0.000255 |
| 19 |  | chr19:19345008 | G | DOM | 2.25 | 1.455 | 3.48 | 0.000266 |
| 12 |  | chr12:111099564 | A | DOM | 2.197 | 1.438 | 3.356 | 0.000271 |
| 3 |  | rs793467 | G | DOM | 2.204 | 1.439 | 3.375 | 0.000281 |
| 4 |  | rs1080081 | G | DOM | 2.186 | 1.432 | 3.335 | 0.000287 |
| 1 |  | chr1:62729773 | A | DOM | 2.26 | 1.454 | 3.512 | 0.000292 |
| 1 |  | chr1:62725969 | A | DOM | 2.256 | 1.452 | 3.506 | 0.000299 |
| 1 |  | chr1:62712752 | A | DOM | 2.252 | 1.449 | 3.5 | 0.00031 |
| 12 |  | rs1796130 | A | DOM | 2.404 | 1.49 | 3.877 | 0.000323 |
| 6 |  | chr6:25793471 | C | DOM | 2.288 | 1.456 | 3.595 | 0.000331 |
| 7 |  | rs17837626 | C | DOM | 2.374 | 1.477 | 3.815 | 0.000354 |
| 10 |  | rs7916162 | A | DOM | 2.368 | 1.474 | 3.805 | 0.000367 |
| 1 |  | chr1:62672716 | A | DOM | 2.305 | 1.455 | 3.653 | 0.000375 |
| 3 |  | rs13070465 | A | DOM | 0.4221 | 0.2625 | 0.679 | 0.000375 |
| 1 |  | chr1:62694201 | T | DOM | 2.219 | 1.428 | 3.449 | 0.000394 |
| 11 |  | chr11:10303570 | C | DOM | 2.408 | 1.48 | 3.917 | 0.000402 |
| 11 |  | chr11:10244386 | A | DOM | 2.126 | 1.397 | 3.234 | 0.000426 |
| 1 |  | chr1:62696498 | G | DOM | 2.209 | 1.421 | 3.432 | 0.000428 |
| 1 |  | chr1:62701709 | G | DOM | 2.207 | 1.419 | 3.433 | 0.000443 |
| 19 |  | rs2910368 | G | DOM | 2.226 | 1.424 | 3.478 | 0.000444 |
| 3 |  | chr3:86129261 | C | DOM | 2.121 | 1.394 | 3.227 | 0.00045 |
| 16 |  | chr16:52509162 | A | DOM | 2.127 | 1.391 | 3.254 | 0.000497 |
| 6 |  | rs4394275 | A | DOM | 0.4617 | 0.2988 | 0.7134 | 0.000499 |
| 19 |  | rs1671152 | A | DOM | 2.239 | 1.42 | 3.531 | 0.000522 |
| 9 |  | rs10984333 | A | DOM | 2.107 | 1.382 | 3.214 | 0.000537 |
| 1 |  | chr1:62678013 | G | DOM | 2.179 | 1.402 | 3.387 | 0.00054 |
| 3 |  | chr3:86133516 | A | DOM | 2.085 | 1.374 | 3.162 | 0.000548 |
| 3 |  | chr3:86133485 | C | DOM | 2.083 | 1.373 | 3.159 | 0.000557 |
| 15 |  | chr15:73056964 | A | DOM | 2.511 | 1.488 | 4.236 | 0.000561 |
| 3 |  | rs6770294 | A | DOM | 0.442 | 0.2777 | 0.7035 | 0.000576 |
| 1 |  | chr1:62702667 | A | DOM | 2.169 | 1.396 | 3.372 | 0.000578 |
| 12 |  | chr12:88541867 | A | DOM | 0.4706 | 0.3062 | 0.7232 | 0.000587 |
| 12 |  | chr12:88490785 | A | DOM | 0.4717 | 0.3072 | 0.7242 | 0.000592 |
| 3 |  | rs11914992 | A | DOM | 2.066 | 1.364 | 3.127 | 0.000608 |
| 11 |  | chr11:10241980 | A | DOM | 2.088 | 1.37 | 3.181 | 0.000612 |
| 4 |  | rs227368 | A | DOM | 2.074 | 1.366 | 3.148 | 0.000615 |
| 6 |  | rs6921497 | A | DOM | 2.227 | 1.408 | 3.522 | 0.000616 |
| 7 |  | chr7:14708236 | G | DOM | 0.3824 | 0.2205 | 0.6631 | 0.000621 |
| 13 |  | rs1547918 | G | DOM | 2.214 | 1.403 | 3.492 | 0.000632 |
| 14 |  | rs10135856 | A | DOM | 0.4776 | 0.3121 | 0.7309 | 0.000664 |
| 8 |  | rs6415517 | A | DOM | 2.506 | 1.476 | 4.254 | 0.000673 |
| 4 |  | rs17028407 | A | DOM | 2.565 | 1.489 | 4.417 | 0.000688 |
| 10 |  | chr10:43838498 | C | DOM | 2.219 | 1.4 | 3.515 | 0.000689 |
| 13 |  | rs9519552 | G | DOM | 2.272 | 1.414 | 3.649 | 0.000691 |
| 10 |  | rs2818912 | G | DOM | 0.4883 | 0.3226 | 0.739 | 0.000699 |
| 6 |  | rs6929849 | A | DOM | 0.4881 | 0.3224 | 0.7389 | 0.000699 |
| 10 |  | chr10:43872248 | A | DOM | 0.4893 | 0.3235 | 0.7401 | 0.00071 |
| 12 |  | rs428073 | G | DOM | 2.078 | 1.359 | 3.176 | 0.000736 |
| 10 |  | chr10:43873589 | A | DOM | 0.4916 | 0.325 | 0.7435 | 0.00077 |
| 11 |  | chr11:10260621 | A | DOM | 2.199 | 1.388 | 3.484 | 0.000785 |
| 5 |  | rs17066506 | A | DOM | 2.127 | 1.369 | 3.305 | 0.000789 |
| 11 |  | chr11:10294686 | A | DOM | 2.32 | 1.417 | 3.799 | 0.000819 |
| 2 |  | rs918290 | A | DOM | 0.4573 | 0.289 | 0.7235 | 0.000831 |
| 11 |  | chr11:10253070 | T | DOM | 2.181 | 1.377 | 3.455 | 0.00089 |
| 11 |  | rs7129220 | A | DOM | 2.309 | 1.41 | 3.784 | 0.000892 |
| 11 |  | chr11:10300557 | A | DOM | 2.303 | 1.407 | 3.768 | 0.000903 |
| 1 |  | rs2422286 | A | DOM | 2.02 | 1.333 | 3.061 | 0.000913 |
| 10 |  | chr10:43987271 | A | DOM | 2.11 | 1.357 | 3.281 | 0.000914 |
| 12 |  | chr12:111122343 | G | DOM | 2.24 | 1.39 | 3.609 | 0.000918 |
| 2 |  | rs12613548 | A | DOM | 0.4443 | 0.2748 | 0.7184 | 0.000936 |
| 6 |  | rs2535315 | A | DOM | 0.4958 | 0.3267 | 0.7525 | 0.00098 |
| 1 |  | chr1:62683859 | C | DOM | 2.111 | 1.353 | 3.293 | 0.000991 |
| 4 |  | rs223482 | A | DOM | 2.15 | 1.363 | 3.392 | 0.000999 |
